# Commentary: Are we always ready for a challenging surprise?

**DOI:** 10.1016/j.xjtc.2021.09.046

**Published:** 2021-09-29

**Authors:** Mindaugas Rackauskas, Thomas Beaver

**Affiliations:** a Division of Thoracic Surgery, Department of Surgery, University of Florida College of Medicine, Gainesville, Fla; b Division of Cardiac Surgery, Department of Surgery, University of Florida College of Medicine, Gainesville, Fla

**Figure:**
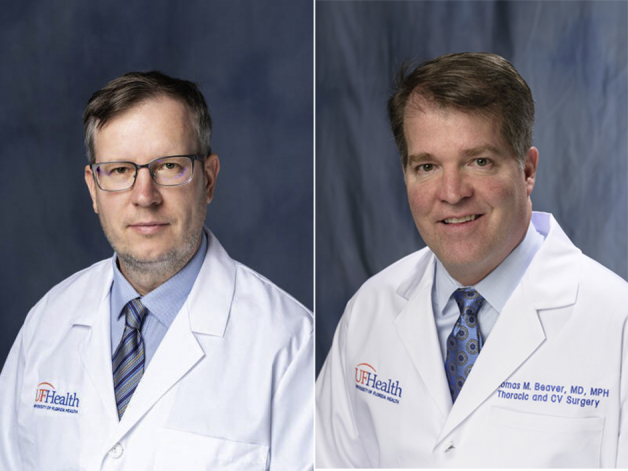
Mindaugas Rackauskas, MD, PhD, and Thomas Beaver, MD, MPH

**Central Message**

Pulmonary artery sarcoma is a rare, but complex pathology requiring multidisciplinary preoperative and postoperative planning, including challenging intraoperative techniques for successful outcomes.

See Article page 317.

Pupovac and colleagues^1^ present an interesting case report of a rarely seen entity, a pulmonary artery sarcoma (PAS). The images shared demonstrate impressive surgical reconstruction with multiple levels of pulmonary artery repair, including the right ventricle outflow tract with an excellent surgical outcome. Unfortunately, the prognosis of patients with PAS usually is poor. The typical cause of death is right-sided heart failure as a result of the right ventricular outflow obstruction.

The clinical presentation of PAS and the radiologic findings are often difficult to interpret, as they can be similar to pulmonary thromboembolism, as in the current case. PAS often presents with symptoms of thromboembolic disease, heart failure, and pulmonary hypertension. Early diagnosis is crucial to perform a curative complete resection. Yeung and colleagues^2^ compared different multimodality imaging, including computed tomography (CT), magnetic resonance imaging, and positron emission tomography, to better characterize PAS. Imaging guides the decision and planning for surgical resection and postoperative therapy. Attina and colleagues^3^ showed that CT characteristics more suggestive of PAS include inhomogeneous high or low attenuation; soft-tissue density with filling defects occupying the entire lumen of the pulmonary trunk along with an increase in diameter of the involved vessel; and patchy and delayed contrast enhancement with CT angiography. Once suspicion is raised, positron emission tomography–CT and magnetic resonance imaging are more helpful to differentiate between sarcoma and pulmonary embolism.

Curative surgical resection of the tumor is not always possible due to advanced disease. Debulking of the obstructive tumor may be chosen to restore hemodynamics, but incomplete resection can lead to early relapse. The primary goal of surgery in PAS is restore the blood flow, decompress the right ventricle, and optimally achieve R0 resection margins. Curative surgical procedures for treating the PAS include tumor endarterectomy, graft reconstruction of the pulmonary artery, and pneumonectomy. In addition, as demonstrated in this report, radical resection of the entire pulmonic root, incorporating pulmonic valve replacement and extensive reconstruction of the right ventricular outflow tract, including combination with multimodality treatment, leads to better survival.

Blackmon and colleagues^4^ found that patients who undergo an attempt at curative resection have longer overall survival compared with those who do not, with median overall survival of 36.5 versus 11 months. Also, the median survival of those who had received multimodality treatment was found to be superior to those who only had single-modality therapy, with median survival of 24.7 versus 8.0 months.

High-volume centers of excellence report similar outcomes, as Wong and colleagues^5^ had median survival of 17 months, with no difference between patients who also underwent pulmonary endarterectomy. Due to the rarity of PAS, so far only small, retrospective case series have been published over the years. This challenging and successful case report with repeated surgical intervention demonstrates how a multidisciplinary approach can lead to improved results.
